# Plasma neutrophil gelatinase-associated lipocalin in acute kidney injury superimposed on chronic kidney disease after cardiac surgery: a multicenter prospective study

**DOI:** 10.1186/cc13104

**Published:** 2013-11-12

**Authors:** Kent Doi, Masahiro Urata, Daisuke Katagiri, Mikako Inamori, Seiichiro Murata, Motoyuki Hisagi, Minoru Ono, Takehiro Matsubara, Takeshi Ishii, Naoki Yahagi, Masaomi Nangaku, Eisei Noiri

**Affiliations:** 1 Department of Emergency and Critical Care Medicine, The University of Tokyo, 7-3-1 Hongo, Bunkyo, Tokyo 113-8655, Japan; 2 Department of Nephrology and Endocrinology, The University of Tokyo, Tokyo, Japan; 3 Department of Cardiothoracic Surgery, The University of Tokyo, Tokyo, Japan; 4 Department of Cardiovascular Surgery, Itabashi Chuo Medical Center, Tokyo, Japan; 5 Japan Science and Technology Agency/Japan International Cooperation Agency (JST/JICA), Science and Technology Research Partnership for Sustainable Development (SATREPS), Tokyo, Japan

## Abstract

**Introduction:**

Plasma neutrophil gelatinase-associated lipocalin (NGAL) is reportedly useful for post-cardiac surgery acute kidney injury (AKI). Although chronic kidney disease (CKD) is a strong risk factor for AKI development, no clinical evaluation of plasma NGAL has specifically examined AKI occurring in patients with CKD. This study evaluated plasma NGAL in AKI superimposed on CKD after cardiac surgery.

**Methods:**

This study prospectively evaluated 146 adult patients with scheduled cardiac surgery at 2 general hospitals. Plasma NGAL was measured before surgery, at ICU arrival after surgery (0 hours), and 2, 4, 12, 24, 36, and 60 hours after ICU arrival.

**Results:**

Based on the Kidney Disease Improving Global Outcomes (KDIGO) CKD guideline, 72 (49.3%) were diagnosed as having CKD. Of 146 patients, 53 (36.3%) developed AKI after surgery. Multiple logistic regression analysis revealed that preoperative plasma NGAL, estimated glomerular filtration rate (eGFR), and operation time are significantly associated with AKI occurrence after surgery. Plasma NGAL in AKI measured after surgery was significantly higher than in non-AKI irrespective of CKD complication. However, transient decrease of plasma NGAL at 0 to 4 hours was observed especially in AKI superimposed on CKD. Plasma NGAL peaked earlier than serum creatinine and at the same time in mild AKI and AKI superimposed on CKD with increased preoperative plasma NGAL (>300 ng/ml). Although AKI superimposed on CKD showed the highest plasma NGAL levels after surgery, plasma NGAL alone was insufficient to discriminate de novo AKI from CKD without AKI after surgery. Receiver operating characteristics analysis revealed different cutoff values of AKI for CKD and non-CKD patients.

**Conclusions:**

Results show the distinct features of plasma NGAL in AKI superimposed on CKD after cardiac surgery: 1) increased preoperative plasma NGAL is an independent risk factor for post-cardiac surgery AKI; 2) plasma NGAL showed an earlier peak than serum creatinine did, indicating that plasma NGAL can predict the recovery of AKI earlier; 3) different cutoff values of post-operative plasma NGAL are necessary to detect AKI superimposed on CKD distinctly from de novo AKI. Further investigation is necessary to confirm these findings because this study examined a small number of patients.

## Introduction

Acute kidney injury (AKI) is a severe complication affecting patients who undergo cardiac surgery. Reportedly, even slight serum creatinine changes during the postoperative period increased mortality in a large cardiac surgery cohort [1]. Nevertheless, the limitations of serum creatinine for the early detection and accurate estimation of renal injury in AKI are well known [2]. Therefore, new AKI biomarkers, such as neutrophil gelatinase-associated lipocalin (NGAL), kidney injury molecule-1 (KIM-1), and L-type fatty acid-binding protein (L-FABP), have been studied intensively in recent years [3-6]. These putatively more sensitive and more accurate biomarkers are also expected to facilitate early intervention before the increase of serum creatinine.

New AKI biomarkers are regarded as having questionable generalizability and reliability in heterogeneous populations before clinical use. During the first clinical evaluations, new AKI biomarkers were examined mostly in pediatric post-cardiac surgery patients, who have fewer comorbid diseases and who show more readily apparent onset of renal insult. In such studies, these new biomarkers showed excellent performance for AKI prediction and detection [7,8]. Several studies have excluded patients with pre-existing renal dysfunction [7-9]. Recently, new AKI biomarkers have shown less impressive performance when tested with more-heterogeneous populations [10]. For adult post-cardiac surgery patients including chronic kidney disease (CKD), urinary NGAL showed a low area under the curve of the receiver operating characteristic curve (AUC (area under the curve)-ROC) of 0.6 to 0.7 [11,12]. Results of several clinical studies addressing baseline renal dysfunction have shown strong effects on the performance of new AKI biomarkers [13,14].

Plasma NGAL, a new AKI biomarker, has demonstrated its clinical utility in studies of both pediatric and adult patients of post-cardiac surgery [12,15-19]. However, as described above, the performance of plasma NGAL for AKI diagnosis was also less impressive in adult post-cardiac surgery patients. Haase-Fielitz and colleagues reported that the performance of serum NGAL was not changed after excluding 27 CKD patients from an adult post-cardiac surgery cohort of 100 patients [16]. However, it remains unclear whether serum NGAL predicted AKI superimposed on CKD in their study. The blood NGAL level is known to be increased in CKD patients under stable conditions [20-22]. Therefore, different cutoff values will be necessary to detect AKI superimposed on CKD distinctly from *de novo* AKI. This study was undertaken to evaluate plasma NGAL as an AKI biomarker with adult post-cardiac surgery patients with or without preoperative complications by CKD.

## Materials and methods

### Patient population

A total of 146 adult patients undergoing scheduled cardiac surgery at Tokyo University Hospital (Tokyo, Japan) and at Itabashi Chuo Medical Center (Tokyo, Japan) were studied prospectively. Patients with end-stage renal disease or a renal transplant were excluded. The study protocol, which adhered to the principles of the Declaration of Helsinki, was approved by the Institutional Review Board of each hospital (The University of Tokyo Institutional Review Board and the Ethics Committee of the Itabashi Chuo Medical Center). Informed consent was obtained from each participant. For each patient, eight blood samples were obtained for serum creatinine measurement, which corresponded to pre-surgery, 0 h (ICU arrival), and 2, 4, 12, 24, 36 and 60 h after ICU arrival.

The presence of AKI was defined by the AKIN (Acute Kidney Injury Network) criteria (an absolute increase in serum creatinine of greater than or equal to 0.3 mg/dl or a percentage increase in serum creatinine of greater than or equal to 50% from the baseline (before surgery)). The AKI severity was also categorized according to the AKIN criteria [23]. Pre-existing chronic kidney disease (CKD) was determined as estimated GFR (eGFR) <60 ml/minute per 1.73 m^2^ or positive kidney damage markers, such as proteinuria (urinary protein of more than 30 mg/g creatinine or dipstick 1+ or more), urine sediment abnormalities and structural abnormalities detected by imaging, or both, according to the KDIGO CKD guideline [24]. These abnormalities of kidney structure or function were present for more than three months. GFR was estimated with the MDRD equation with a known baseline creatinine value [25]. Two board-certified nephrologists (KD and DK) independently reviewed all medical records and confirmed the determination of CKD.

### Plasma NGAL measurement

Plasma NGAL was determined at the same time points of serum creatinine described above (pre-surgery, 0, 2, 4, 12, 24, 36 and 60 h after ICU arrival) using an NGAL test (Triage; Alere Medical Inc., San Diego, CA, USA). The test is a point-of-care, fluorescence-based immunoassay designed for rapid quantitative measurement of NGAL in EDTA-anticoagulated whole blood. Moreover, the extended-range sandwich test was used for this study [26]. This assay was developed for the larger quantifiable range of NGAL compared with the existing competitive assay. The antibodies used in the sandwich immunoassay were selected to target only the free form of NGAL, not NGAL in the homodimeric form or heterodimeric complexes with matrix metallopeptidase 9.

### Adjustment of serum creatinine and plasma NGAL by body weight gain

Adjustment of serum creatinine and plasma NGAL was conducted as described previously [27,28]. Adjusted values were calculated as follows: adjusted serum creatinine/plasma NGAL = serum creatinine/plasma NGAL × (1+ (postoperative body weight gain/0.6 × preoperative body weight)).

### Statistical analyses

Data were expressed as median (interquartile). Continuous variables were compared using *t*-tests or Wilcoxon rank-sum tests when the normality assumption does not hold. The Tukey–Kramer or Steel-Dwass test were used for multiple comparison. Categorical variables were compared using the Pearson χ^2^ or Fisher’s exact test. The performance of urinary biomarkers was determined using receiver operating characteristic (ROC) curve analysis. Optimal cutoff values were determined using the Youden index (sensitivity + specificity - 1). This index is a common summary measure of the ROC curve, representing the maximum potential effectiveness of a marker [29]. Cutoff values that provide 95% sensitivity or 95% specificity were also calculated. A meta-analysis reported the AUC-ROC value of NGAL for AKI after cardiac surgery was 0.78 (95% confidence interval 0.67 to 0.87) [30]. We estimated that a sample size of 129 patients would be necessary to detect a significant difference in AUC-ROC of 0.65 vs. 0.50 (null hypothesis) with the ratio of sample size in negative/positive 2.0 at 80% power and at a significance level of 0.05. Wilcoxon signed-rank test was used to compare the postoperative values of serum creatinine and plasma NGAL with those measured before surgery. These calculations were performed using software (JMP ver. 9.0; SAS Institute Inc., Cary, NC, USA and MedCalc Version 12.7.1.0; MedCalc Software, Ostend, Belgium). A conventional criterion of an alpha level of 0.05 was used to determine statistical significance.

## Results

### Patient characteristics and preoperative plasma NGAL

**Figure 1:**
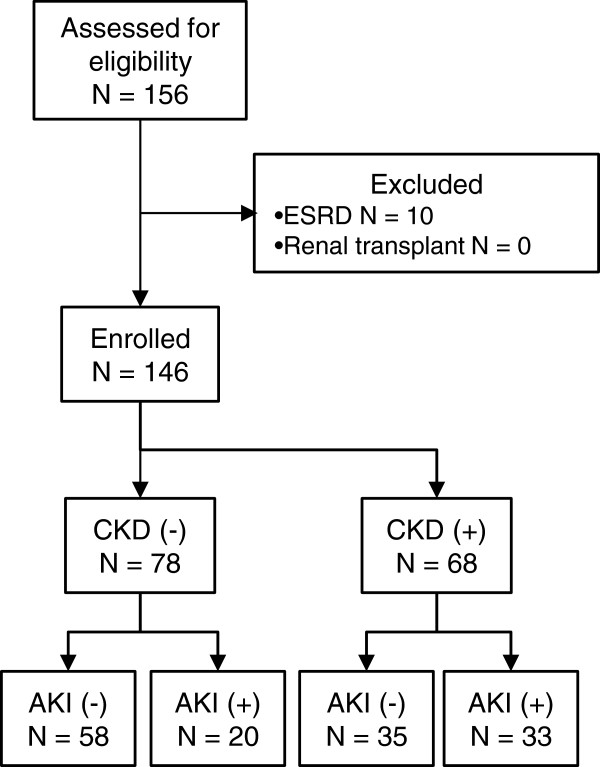
**Study flow diagram.** Acute kidney injury (AKI) and chronic kidney disease (CKD) were defined using the AKIN and KDIGO CKD guideline criteria.

This study prospectively analyzed 146 adult patients who had scheduled cardiac surgery at two general hospitals. Of those, 68 patients (47.6%) were diagnosed as having CKD defined by the KDIGO guideline [24]. AKI was diagnosed using the serum creatinine criteria of AKIN (that is, a minimum of 0.3 mg/dl or 50% increase in serum creatinine from the baseline measured before surgery). Of 146 adult post-cardiac surgery patients, AKI was diagnosed in 53 (36.3%) within three days after surgery (AKIN stage 1, n = 39; stage 2, n = 5; stage 3, n = 9). Among 53 AKI patients, 24 patients were diagnosed as having AKI by a minimum of 0.3 mg/dl increase in serum creatinine. These patients mostly showed mild AKI (AKIN stage 1, n = 22), although two patients eventually required renal replacement therapy (stage 3, n = 2). Table 1 and Figure 1 present the prevalence of AKI and CKD, baseline clinical data, surgical procedures and outcomes of the enrolled patients in this study.

**Table 1:** Patient characteristics and clinical outcomes

	**Non-CKD, Non-AKI**	**Non-CKD, AKI**	**CKD, Non-AKI**	**CKD, AKI**	***P-*** **value**
	**(n = 58)**	**(n = 20)**	**(n = 35)**	**(n = 33)**	
Age (y.o.)	67.5 (57.8 to 74.0)	64.5 (53.3 to 73.0)	74.0 (69.0 to 79.0)	69.0 (65.5 to 76.0)	0.0005
Male, *n* (%)	36 (62.1%)	16 (80.0%)	23 (65.7%)	17 (51.5%)	0.2
Diabetes, *n* (%)	24 (41.4%)	8 (40.0%)	15 (42.9%)	12 (36.4%)	0.95
Hypertension, *n* (%)	34 (58.6%)	15 (75.0%)	24 (68.6%)	22 (66.7%)	0.54
Preoperative					
Serum Cre (mg/dl)	0.75 (0.61 to 0.83)	0.76 (0.67 to 0.89)	1.13 (1.01 to 1.38)	1.39 (1.01 to 1.96)	<0.0001
eGFR (ml/min/1.73 m^2^)	75.0 (67.2 to 82.1)	71.8 (64.1 to 83.6)	48.5 (35.3 to 53.4)	36.7 (26.1 to 53.0)	<0.0001
Plasma NGAL (ng/ml)	61.0 (42.2 to 2.4)	75.5 (61.0 to 121.0)	120.0 (66.7 to 224.0)	209.0 (106.5 to 492.0)	<0.0001
Operation					
Operation time (minutes)	296 (259 to 358)	330 (280 to 478)	304 (257 to 362)	355 (289 to 420)	0.064
OPCAB, *n* (%)	11 (19.0%)	5 (25.0%)	10 (28.6%)	6 (18.2%)	0.67
CPB, *n* (%)	47 (81.0%)	15 (75.0%)	25 (71.4%)	27 (81.8)	0.67
CPB time (minutes)	151 (119 to 204)	168 (132 to 286)	149 (115 to 195)	170 (110 to 228)	0.4
Valve surgery, *n* (%)	17 (29.3%)	7 (35.0%)	15 (42.9%)	12 (36.4%)	0.62
CABG surgery, *n* (%)	7 (12.1%)	1 (5.0%)	1 (2.9%)	5 (15.2%)	0.27
Valve + CABG, *n* (%)	7 (12.1%)	4 (20.0%)	5 (14.3%)	5 (15.2%)	0.86
Aortic graft replacement, *n* (%)	12 (20.7%)	3 (15.0%)	2 (5.7%)	1 (3.0%)	0.05
Other, *n* (%)	4 (6.9%)	0 (0.0%)	2 (5.7%)	4 (12.1%)	0.48
Postoperative					
Body weight gain (kg)	1.95 (0.77 to 3.30)	2.96 (1.33 to 4.73)	2.04 (1.07 to 3.93)	2.45 (0.64 to 3.88)	0.66
Need for RRT, *n* (%)	0 (0.0%)	2 (10.0%)	0 (0.0%)	7 (21.2%)	0.0001
ICU stay (day)	3 (2 to 4)	4 (2 to 10)	3 (2 to 4)	4 (2 to 10)	0.0077

CABG, coronary artery bypass grafting; CKD, chronic kidney disease; CPB, cardiopulmonary bypass; OPCAB, off-pump coronary artery bypass grafting; RRT, renal replacement therapy.

Serum creatinine and plasma NGAL before surgery in the AKI group was significantly higher than in the non-AKI group (1.00 (0.80 to 1.52) mg/dl vs. 0.80 (0.67 to 1.06) mg/dl, *P* = 0.0004). AKI was observed with greater relative frequency in the CKD patients than in non-CKD patients (33 of 68 CKD patients (48.5%) vs. 20 of 78 non-CKD (25.6%), odds ratio 2.73, 95% CI 1.36 to 5.48). A significant correlation was found between estimated GFR and plasma NGAL measured before surgery (*R*^2^ = 0.254, *P* <0.0001). Although no difference was found in the frequency of cardiopulmonary bypass (CPB) between the AKI group and the non-AKI group, the operation time and CPB time in the AKI group were significantly longer than in the non-AKI group. The length of ICU stay in the AKI group was significantly longer. Moreover, renal replacement therapy was necessary only for AKI patients.

A multiple logistic regression analysis incorporating parameters with a univariate *P-*value <0.05 (age, eGFR, operation time and plasma NGAL before surgery) revealed that plasma NGAL before surgery was independently associated with post-surgery AKI occurrence (Table 2).

**Table 2:** Multiple logistic analysis for AKI diagnosis

**Variable**	**Regression coefficient (95% confidence interval)**	***P-*** **value**
Age	-0.025 (-0.065 to 0.015)	0.22
eGFR	-0.025 (-0.048 to -0.003)	0.03
Operation time (minutes)	0.004 (0.001 to 0.008)	0.03
Plasma NGAL before surgery (ng/ml)	0.003 (0.001 to 0.007)	0.03

AKI, acute kidney injury; eGFR, estimated glomerular filtration rate; NGAL, neutrophil gelatinase-associated lipocalin.

### Postoperative plasma NGAL for detection of AKI, and prediction of recovery and dialysis requirement

**Figure 2:**
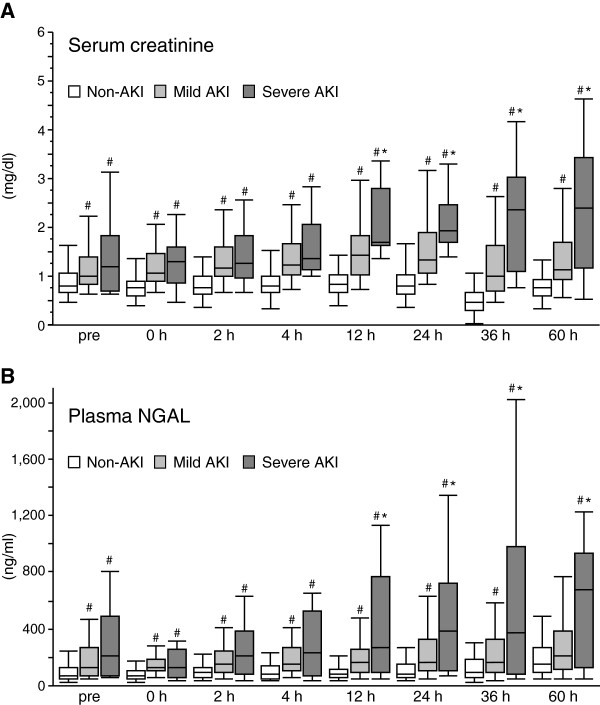
**Serum creatinine and plasma NGAL values grouped by AKI severity.** Values of serum creatinine **(A)** and plasma neutrophil gelatinase-associated lipocalin (NGAL) **(B)** for acute kidney injury (AKI) severity categories (No AKI (n = 89), Mild AKI (n = 39), Severe AKI (n = 14)). #, *P* <0.05 vs. non-AKI; *, *P* <0.05 vs. mild AKI.

Plasma NGAL was measured before surgery, and at 0, 2, 4, 12, 24 and 36 h after ICU arrival. Plasma NGAL levels in the AKI group were significantly higher than in the non-AKI group at all time points. At 12 h and thereafter, plasma NGAL levels in the non-AKI, mild AKI (AKIN stage 1), and severe AKI (AKIN stage 2 + 3) groups were significantly different from each other, indicating that plasma NGAL can reflect the AKI severity (Figure 2). Nine of 53 AKI patients required dialysis 12 h after surgery or thereafter. These patients showed significantly higher plasma NGAL levels before and at 0 and 4 h after the surgery than non-dialysis requiring AKI patients showed (Table 3).

**Table 3:** ROC analysis of plasma NGAL for dialysis requiring AKI

	**Non-dialysis requiring AKI**	**Dialysis requiring AKI**	**AUC-ROC**	**Cutoff (Youden)**	**Sensitivity (Youden)**	**Specificity (Youden)**	**Cutoff (95% sensitivity)**	**Specificity (95% sensitivity)**	**Cutoff (95% specificity)**	**Sensitivity (95% specificity)**
	**(n = 44)**	**(n = 9)**	**(95% CI)**	**(ng/ml)**			**(ng/ml)**		**(ng/ml)**	
pre	122.5 (71.3 to 241.5)	482.0 (178.4 to 531.0)#	0.77	472	67%	89%	75	30%	785	11%
			(0.55 to 0.90)							
0 h	125.0 (90.0 to 187.8)	162.0 (121.0 to 272.0)	0.66	254	44%	89%	61	5%	469	0%
			(0.44 to 0.83)							
2 h	143.0 (89.7 to 238.8)	268.0 (171.0 to 419.0)#	0.75	170	89%	61%	89	23%	861	0%
			(0.54 to 0.88)							
4 h	152.0 (92.9 to 228.8)	383.0 (235.0 to 619.5)#	0.81	220	89%	75%	100	27%	608	28%

AKI, acute kidney injury; AUC-ROC, area under the curve operating characteristic curves; CI, confidence interval; NGAL, neutrophil gelatinase-associated lipocalin.

**Figure 3:**
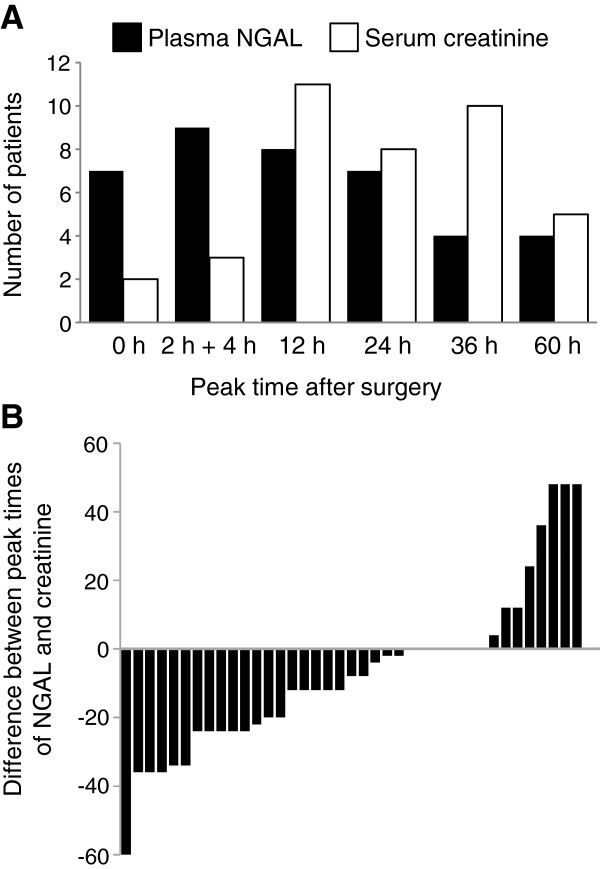
**Peak time of plasma NGAL and serum creatinine. (A)** Time points of the highest plasma neutrophil gelatinase-associated lipocalin (NGAL) and serum creatinine values in mild acute kidney injury (AKI) patients (n = 39). **(B)** Time lags of the peaks between plasma NGAL and serum creatinine in each AKI patient. Negative values indicate plasma NGAL started to decrease earlier than serum creatinine did. Serum creatinine started to decrease earlier than plasma NGAL in only 8 of 39 mild AKI patients (21%).

Evaluation of plasma NGAL for predicting AKI recovery was conducted in the mild AKI group (n = 39), in which no patient either died or required dialysis. Plasma NGAL showed its highest values earlier than serum creatinine did (Figure 3A). The temporal difference between the peaks of plasma NGAL and serum creatinine was -8 (-24 to 0) h (Figure 3B).

### Differences between de novo AKI and AKI superimposed on CKD

**Figure 4:**
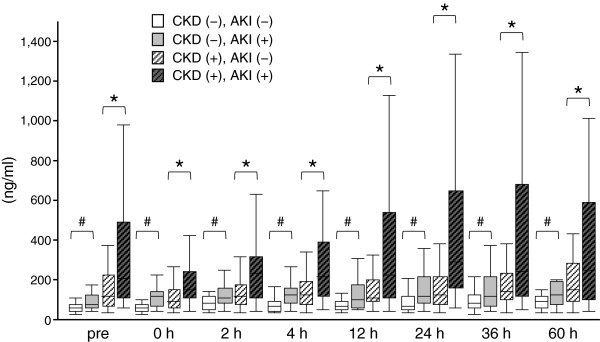
**Plasma NGAL values in *de novo *AKI and AKI superimposed on CKD*.*** Values of plasma neutrophil gelatinase-associated lipocalin (NGAL) for four groups (CKD(-)AKI(-) (n = 54), CKD(-)AKI(+) (n = 20), CKD(+)AKI(-) (n = 39), and CKD(+)AKI(+) (n = 33)). #, *P* <0.05 vs. CKD(-)AKI(-); *, *P* <0.05 vs. CKD(+)AKI(-). AKI, acute kidney injury; CKD, chronic kidney disease.

The patients were divided into four groups to clarify the impact of baseline renal dysfunction as follows: CKD(-)AKI(-) (n = 54), CKD(-)AKI(+) (n = 20), CKD(+)AKI(-) (n = 39), and CKD(+)AKI(+) (n = 33). Plasma NGAL levels before surgery were significantly higher in the CKD(-)AKI(+) group than in the CKD(-)AKI(-) group, and were also higher in the CKD(+)AKI(+) group than in the CKD(+)AKI(-) group (Table 1 and Figure 4). Plasma NGAL values measured before surgery in the CKD(+)AKI(+) group were the highest among these four groups. However, for eGFR no significant difference was found between the CKD(+)AKI(+) group and the CKD(+)AKI(-) group (37.5 ± 14.2 vs. 45.8 ± 10.2 ml/minute per 1.73 m^2^, *P* = 0.0716) or between the CKD(-)AKI(+) group and the CKD(-)AKI(-) group (75.0 ± 13.8 vs. 80.2 ± 16.7 ml/minute per 1.73 m^2^, *P* = 0.5097). This result is consistent with the results of multiple logistic analyses that both eGFR and plasma NGAL measured before surgery were independently associated with postoperative AKI occurrence (Table 2).

After surgery, AKI patients with or without CKD complication showed significantly higher plasma NGAL values than non-AKI patients did. However, the CKD(-)AKI(+) and the CKD(+)AKI(-) group showed similar plasma NGAL values for all perioperative sampling points (Figure 4). Results of ROC analysis are presented in Table 4 and Additional file 1: Figure S1. Plasma NGAL showed fair performance for AKI diagnosis, with AUC-ROC values above 0.70. It is noteworthy that the cutoff values of AKI prediction in CKD were higher than those of non-CKD at all time-points.

**Table 4:** ROC analysis of plasma NGAL in non-CKD and CKD patients

**Non-CKD**	**AUC-ROC**	**Cutoff (Youden)**	**Sensitivity (Youden)**	**Specificity (Youden)**	**Cutoff (95% sensitivity)**	**Specificity (95% sensitivity)**	**Cutoff (95% specificity)**	**Sensitivity (95% specificity)**
**(n = 78)**	**(95% CI)**	**(ng/ml)**			**(ng/ml)**		**(ng/ml)**	
pre	0.69	73	65%	75%	52	33%	298	15%
	(0.55 to 0.81)							
0 h	0.76	102	65%	90%	49	33%	147	23%
	(0.60 to 0.87)							
2 h	0.67	142	45%	90%	48	19%	220	13%
	(0.50 to 0.80)							
4 h	0.76	72	90%	60%	49	28%	226	15%
	(0.60 to 0.86)							
12 h	0.73	90	70%	76%	48	26%	226	15%
	(0.56 to 0.85)							
24 h	0.73	91	75%	66%	47	14%	240	20%
	(0.57 to 0.84)							
36 h	0.63	113	55%	72%	44	10%	376	5%
	(0.46 to 0.77)							
60 h	0.62	124	55%	77%	35	1%	284	20%
	(0.43 to 0.77)							
**CKD**	**AUC-ROC**	**Cutoff (Youden)**	**Sensitivity (Youden)**	**Specificity (Youden)**	**Cutoff (95% sensitivity)**	**Specificity (95% sensitivity)**	**Cutoff (95% specificity)**	**Sensitivity (95% specificity)**
**(n = 68)**	**(95% CI)**	**(ng/ml)**			**(ng/ml)**		**(ng/ml)**	
pre	0.71	472	33%	100%	68	23%	341	33%
	(0.56 to 0.82)							
0 h	0.75	111	76%	63%	77	32%	233	30%
	(0.61 to 0.85)							
2 h	0.75	233	52%	94%	59	16%	241	44%
	(0.61 to 0.85)							
4 h	0.74	163	70%	69%	69	16%	286	40%
	(0.60 to 0.84)							
12 h	0.72	340	42%	97%	71	19%	322	42%
	(0.58 to 0.83)							
24 h	0.77	241	59%	85%	70	24%	363	43%
	(0.63 to 0.87)							
36 h	0.71	373	45%	94%	71	11%	379	44%
	(0.56 to 0.82)							
60 h	0.66	537	31%	97%	62	10%	432	31%
	(0.51 to 0.78)							

AUC-ROC, area under the curve operating characteristic curves; CI, confidence interval; CKD, chronic kidney disease; NGAL, neutrophil gelatinase-associated lipocalin.

### Transient decrease of plasma NGAL in AKI superimposed on CKD

Increased preoperative plasma NGAL in the CKD(+)AKI(+) group was followed by a transient drop at 0 to 4 h after the surgery (Figure 4). However, the patients of CKD(+) AKI(+) whose pre-operative plasma NGAL had already increased before the surgery (>300 ng/ml) showed further elevation of plasma NGAL 12 h after the surgery and thereafter (Additional file 2: Figure S2B). In these patients, the highest value of postoperative plasma NGAL was observed earlier than serum creatinine (Additional file 2: Figure S2C, D) as observed in mild AKI patients (Figure 3). Intraoperative fluid administration can cause dilution of blood concentrations of creatinine and NGAL. Therefore, we examined whether fluid accumulation decreased serum creatinine and plasma NGAL by adjustment with body weight gain after surgery (Additional file 3: Figure S3). Even after adjustment for fluid accumulation, a transient drop of plasma NGAL at 0 h was observed in the CKD(+)AKI(+) and the CKD(-)AKI(+) groups.

## Discussion

This study evaluated detection of AKI superimposed on CKD by plasma NGAL measurement in adult post-cardiac surgery patients. Reports of two large clinical observational studies described that CKD was found in approximately 30% of AKI patients in the ICU [31,32]. Nash and colleagues reported that patients with underlying CKD were approximately three times more likely to develop AKI than were patients with normal renal function [33]. Results of this study also clarified that CKD was associated significantly with AKI occurrence after cardiac surgery. Hsu and colleagues analyzed a large community-based cohort of patients with CKD and reported that AKI superimposed on CKD is a strong indicator of risk for death or end-stage renal disease (ESRD) [34]. Therefore, an AKI biomarker that can detect both *de novo* AKI and AKI superimposed on CKD is expected to be useful in a clinical setting.

In this study, elevation of plasma NGAL was found to be an independent risk factor for AKI occurrence after surgery in addition to preoperative eGFR (Table 2). This finding suggests that measurement of plasma NGAL in CKD before surgery will enable identification of high-risk populations of AKI superimposed on CKD, a strong predictor of death or ESRD. It is noteworthy that nearly 50% of patients in the present cohort were complicated with CKD before surgery, whereas previous clinical evaluation studies of plasma NGAL on adult post-cardiac surgery AKI excluded CKD patients or did not analyze the impact of pre-existing CKD on the performance of plasma NGAL [12,16,17].

Not only early detection of AKI but early prediction of recovery is an important factor for the AKI biomarker because serum creatinine cannot respond quickly to renal recovery in AKI. In this study, plasma NGAL started to decrease earlier than serum creatinine did (Figures 3 and S2). In addition to increased mortality, mild AKI defined as small serum creatinine elevation (>0.3 mg/dl or a 50%) will cause delayed ICU discharge and a subsequently longer stay in the ICU. A biomarker that can predict the recovery of AKI earlier than serum creatinine is expected to be helpful in determining ICU discharge.

Reportedly, blood NGAL levels are inversely and closely related to eGFR in stable CKD patients [20-22]. Results of the present study also show a significant, negative correlation between eGFR and plasma NGAL measured before surgery. These data suggest that *de novo* AKI and AKI superimposed on CKD have different cutoff values. When we classified the patients into four groups using AKI and pre-existing CKD, the CKD(-)AKI(+) group and the CKD(+)AKI(-) group showed similar plasma NGAL levels at all perioperative sampling points (Figure 4). Therefore, plasma NGAL levels should be interpreted with the existence of preoperative CKD. This interpretation might be consistent with a report by Endre and colleagues, who described the improvement of new urinary AKI biomarkers, including NGAL, cystatin C, KIM-1 and interleukin-18 by stratification with baseline renal function [14]. Actually, ROC analysis in this study revealed that the cutoff values of plasma NGAL for AKI defined by the Youden index in the non-CKD patients were all below 150 ng/ml, whereas results obtained with the CKD patients showed cutoff values that were mostly greater than 150 ng/ml. It is quite possible that the performance of plasma NGAL would be reduced without consideration of baseline renal function and without application of a proper threshold value for AKI diagnosis. Haase-Fielitz and colleagues measured serum NGAL in an adult cardiac surgery cohort at a single center (n = 100; CKD 27%). They observed no preoperative difference of serum creatinine or serum NGAL between AKI and non-AKI patients [16]. Further investigation with a larger sample size in a multicenter study is necessary to ascertain the impact of pre-existing CKD on plasma and serum NGAL levels before introducing NGAL measurement to clinical use.

Although preoperative plasma NGAL was useful to identify the high-risk patients for AKI after the surgery (Table 2), increased NGAL from preoperative values will not enable us to detect AKI early after the surgery because of its transient decrease at 0 to 4 h, especially in the CKD patients. However, the patients of CKD(+) AKI(+) whose pre-operative plasma NGAL was already increased before the surgery (>300 ng/ml) showed further elevation of plasma NGAL 12 h after the surgery and thereafter (Additional file 2: Figure S2B). In addition, the highest value of postoperative plasma NGAL was observed earlier than that of serum creatinine not only in mild AKI (Figure 3) but in these CKD(+)AKI(+) patients (Additional file 2: Figure S2C, D). These observations suggest that plasma NGAL is useful not for early AKI detection but for predicting the severity of AKI earlier than serum creatinine. We calculated the adjusted blood creatinine and NGAL levels at 0 h by considering the impact of fluid accumulation during surgery (Additional file 3: Figure S3). Even after adjustment for fluid accumulation, transient decreases of plasma NGAL but not serum creatinine at 0 to 4 h after the surgery was observed in the CKD(+)AKI(+) group. This result indicates another unknown mechanism that causes transient suppression of plasma NGAL in CKD induced by cardiac surgery rather than dilution. Further evaluation is necessary to confirm these observations.

Cai and colleagues reported that several different molecular forms of NGAL were found in human urine and that the monometric form is predominantly secreted by cultured renal tubular epithelial cells, whereas the dimeric form is predominantly secreted by neutrophils [35]. NGAL was first characterized as a protein complexed with metallopeptidase 9 (MMP-9) released from stimulated neutrophil [36]. Therefore, it is important to clarify the measured forms of NGAL by the assay. This study used sandwich format immunoassay. The antibodies that were used recognize only the free form of NGAL, not NGAL in the homodimeric form or heterodimeric complexes with MMP-9 [26].

Several limitations possibly affected the results obtained in this study. First, although the patients were enrolled at two general hospitals, their number (n = 146) might be insufficient to determine the reliability and generalizability of plasma NGAL. Recently, a large multicenter cohort study (TRIBE-AKI) of 1,219 adult patients undergoing cardiac surgery evaluating urinary IL-18, urinary NGAL, or plasma NGAL was reported [37]. Thirty-five percent of this cohort had CKD (eGFR <60). The discriminatory ability of plasma NGAL did not differ by CKD. It is noteworthy that only 60 patients developed AKI, as defined differently from the present study (requiring acute dialysis or doubling of serum creatinine after surgery). Second, AKI was diagnosed only with serum creatinine. Although the AKIN criteria suggest the use of another criterion based on urine output, recent studies have frequently employed the serum creatinine-based criterion alone [38]. Finally, AKI diagnosis based on serum creatinine might underestimate renal injury. It has been suggested that a composite endpoint by clinical events other than a short-term change in serum creatinine should be regarded as a major adverse kidney event. A multicenter pooled analysis of NGAL in AKI revealed that the subgroup of increased NGAL with no serum creatinine elevation (NGAL-positive creatinine-negative) had adverse clinical outcomes including mortality, dialysis requirement, ICU stay and overall hospital stay [39]. Recently, the 10th Consensus Conference of the Acute Dialysis Quality Initiative (ADQI) reported a combination of kidney functional (serum creatinine and urine volume) and damage markers (new biomarkers including NGAL) to stratify patients with AKI [40]. AKI can be diagnosed only using damage markers, such as NGAL, even when no change in serum creatinine or urine volume is observed (structural AKI). Pickering and colleagues evaluated the performance of plasma NGAL on structural AKI defined by increased urine NGAL with an adult ICU cohort [41], although this study did not evaluate the performance of plasma NGAL on creatinine-independent diagnosis of AKI occurred in a different cohort of post-cardiac surgery. Additional evaluation must be undertaken to clarify the role of plasma NGAL for detecting structural AKI that would be diagnosed independently from serum creatinine values.

## Conclusions

This study demonstrated that preoperative plasma NGAL measurement is useful for identifying a high-risk population of AKI. Although a transient decrease of plasma NGAL occurred immediately after surgery (0 to 4 h), especially in AKI superimposed on CKD, plasma NGAL showed an earlier peak than did serum creatinine, indicating that plasma NGAL can predict the recovery of AKI earlier. Moreover, determining different cutoff values of postoperative plasma NGAL for *de novo* AKI and AKI superimposed on CKD was necessary for accurate AKI diagnosis. Further investigation is necessary to confirm these findings because this study enrolled a small number of patients.

## Key messages

•This study evaluates plasma NGAL in AKI occurring in patients with and without CKD after cardiac surgery because CKD is a strong risk factor for AKI development.

•Preoperative plasma NGAL, estimated GFR and operation time are independently and significantly associated with AKI occurrence after surgery.

•Although AKI superimposed on CKD showed the highest plasma NGAL levels after surgery, plasma NGAL alone was insufficient to discriminate *de novo* AKI from CKD without AKI after surgery.

•Plasma NGAL showed an earlier peak than serum creatinine did, indicating that plasma NGAL can predict the recovery of AKI earlier.

## Abbreviations

AKI: Acute kidney injury; AUC: Area under the curve; CABG: Coronary artery bypass grafting; CKD: Chronic kidney disease; CPB: Cardiopulmonary bypass; ESRD: End-stage renal disease; GFR: Glomerular filtration rate; ICU: Intensive care unit; KIM-1: Kidney injury molecule-1; L-FABP: L-type fatty acid-binding protein; NGAL: Neutrophil gelatinase-associated lipocalin; OPCAB: Off-pump coronary artery bypass grafting; ROC: Receiver operating characteristics; RRT: Renal replacement therapy.

## Competing interests

The authors declare that they have no competing interests.

## Authors’ contributions

KD and MU conceived of the study, participated in its design and coordination, conducted sample collection, measured biomarkers, analyzed the data, and drafted the manuscript. DK and MI conducted sample collection and measured biomarkers. SM participated in its design and coordination, analyzed the data, and drafted the manuscript. MH conducted sample collection and measured biomarkers. MO conceived of the study, participated in its design and coordination, analyzed the data, and drafted the manuscript. TM participated in its design and coordination, conducted sample collection and measured biomarkers. TI participated in its design and coordination, conducted sample collection and measured biomarkers. NY and MN conceived of the study, participated in its design and coordination, analyzed the data, and drafted the manuscript. EN conceived of the study and participated in its design and coordination. KD and MU equally contributed to this study. All authors read and approved the final manuscript.

## Supplementary Material

Additional file 1: Figure S1 The following additional data are available with the online version of this paper. **ROC analysis for AKI diagnosis at each time point.** Among 143 enrolled patients, 68 patients were diagnosed as having CKD and 78 were not (non-CKD). ROC curves for AKI diagnosis in non-CKD (A-H) and CKD (I-P) are shown. Three cutoff values determined by Youden index, 95% sensitivity and 95% specificity are illustrated on the curve. The AUC-ROC values are presented in Table 3. Click here for file

Additional file 2: Figure S2 The following additional data are available with the online version of this paper. **Time course of plasma NGAL and serum creatinine in AKI superimposed on CKD.** The CKD(+)AKI(+) group was divided into two groups by preoperative plasma NGAL values (Pre NGAL <300 ng/ml (n = 22) and Pre NGAL >300 ng/ml (n = 11)). Values of serum creatinine **(A)** and plasma NGAL **(B)** in each group are shown. **(C)** Time points of the highest plasma NGAL and serum creatinine values in the Pre NGAL >300 ng/ml group (n = 11). **(D)** Time lags of the peaks between plasma NGAL and serum creatinine in each AKI patient. Negative values indicate plasma NGAL started to decrease earlier than serum creatinine. #, *P* <0.05 vs. before surgery (pre). Click here for file

Additional file 3: Figure S3 The following additional data are available with the online version of this paper. **Dilution effect on serum creatinine and plasma NGAL at 0 h.** Values of serum creatinine **(A)** and plasma NGAL **(B)** before surgery and at 0 h (with and without adjustment by fluid accumulation) are shown (CKD(-)AKI(-) (n = 54), CKD(-)AKI(+) (n = 20), CKD(+)AKI(-) (n = 39), and CKD(+)AKI(+) (n = 33)). #, *P* <0.05 vs. before surgery (pre), *, *P* <0.05 vs. 0 h. Click here for file
