# Supplementary figures and images for: Plasma neutrophil gelatinase-associated lipocalin in acute kidney injury superimposed on chronic kidney disease after cardiac surgery: a multicenter prospective study
Source: Crit Care. 2013 Nov 12;17(6):R270. doi: 10.1186/cc13104 (PMC4056897; doi:10.1186/cc13104)

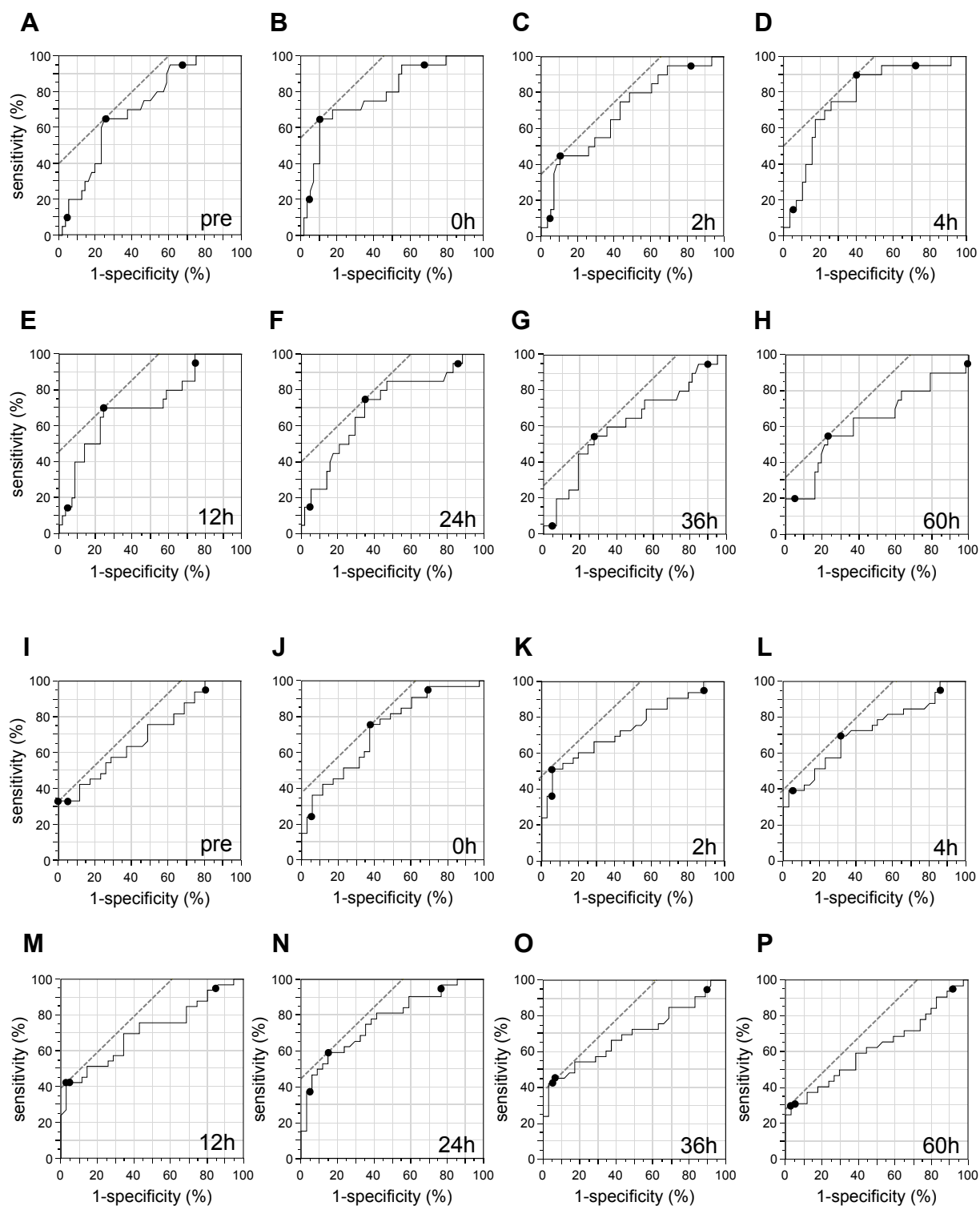

Supplementary figure 1

Supplement: Additional file 1: Figure S1 — The following additional data are available with the online version of this paper. ROC analysis for AKI diagnosis at each time point. Among 143 enrolled patients, 68 patients were diagnosed as having CKD and 78 were not (non-CKD). ROC curves for AKI diagnosis in non-CKD (A-H) and CKD (I-P) are shown. Three cutoff values determined by Youden index, 95% sensitivity and 95% specificity are illustrated on the curve. The AUC-ROC values are presented in Table 3. [file cc13104-S1.pdf]

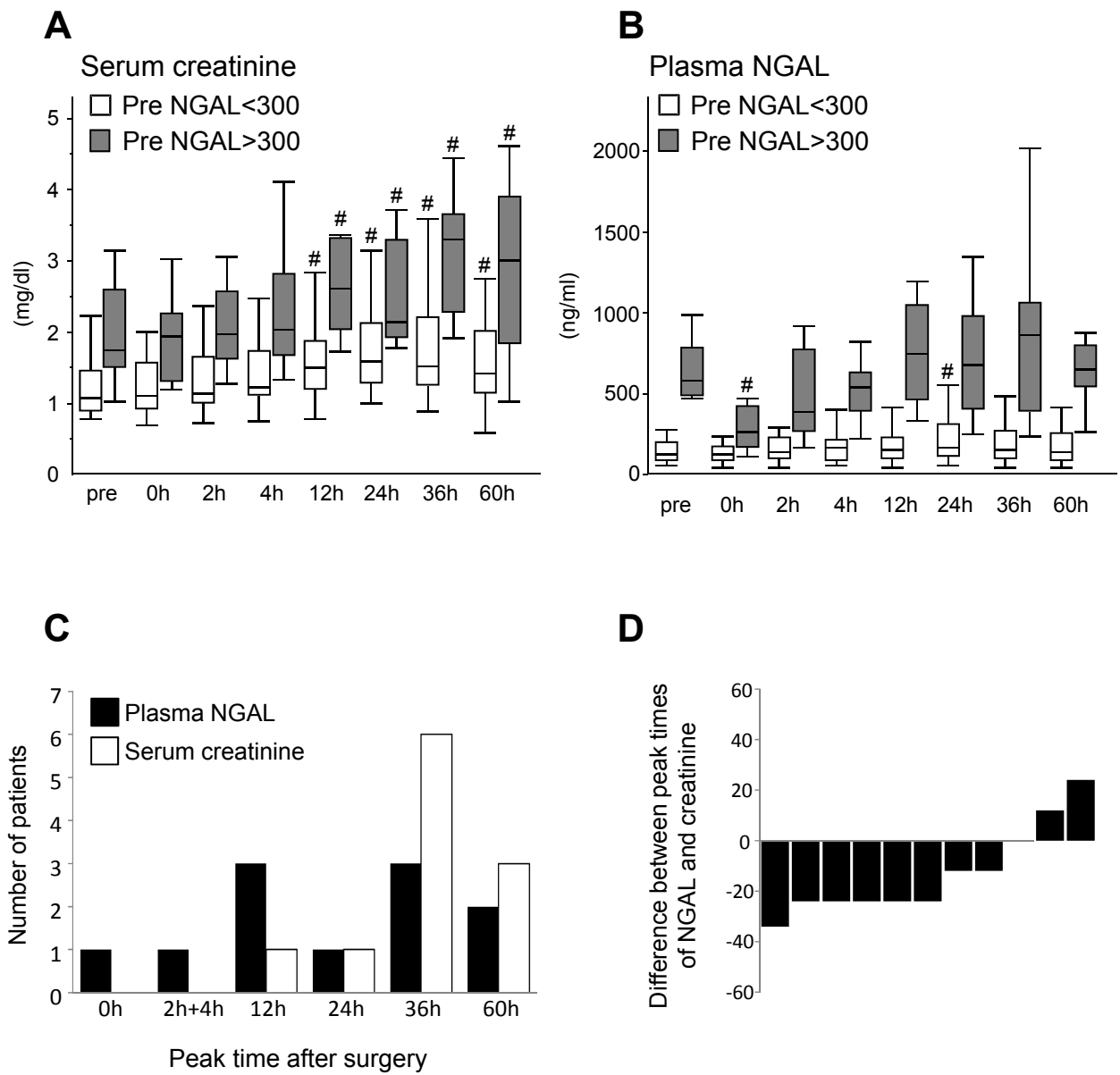

Supplementary figure 2

Supplement: Additional file 2: Figure S2 — The following additional data are available with the online version of this paper. Time course of plasma NGAL and serum creatinine in AKI superimposed on CKD. The CKD(+)AKI(+) group was divided into two groups by preoperative plasma NGAL values (Pre NGAL <300 ng/ml (n = 22) and Pre NGAL >300 ng/ml (n = 11)). Values of serum creatinine (A) and plasma NGAL (B) in each group are shown. (C) Time points of the highest plasma NGAL and serum creatinine values in the Pre NGAL >300 ng/ml group (n = 11). (D) Time lags of the peaks between plasma NGAL and serum creatinine in each AKI patient. Negative values indicate plasma NGAL started to decrease earlier than serum creatinine. #, P <0.05 vs. before surgery (pre). [file cc13104-S2.pdf]

**A**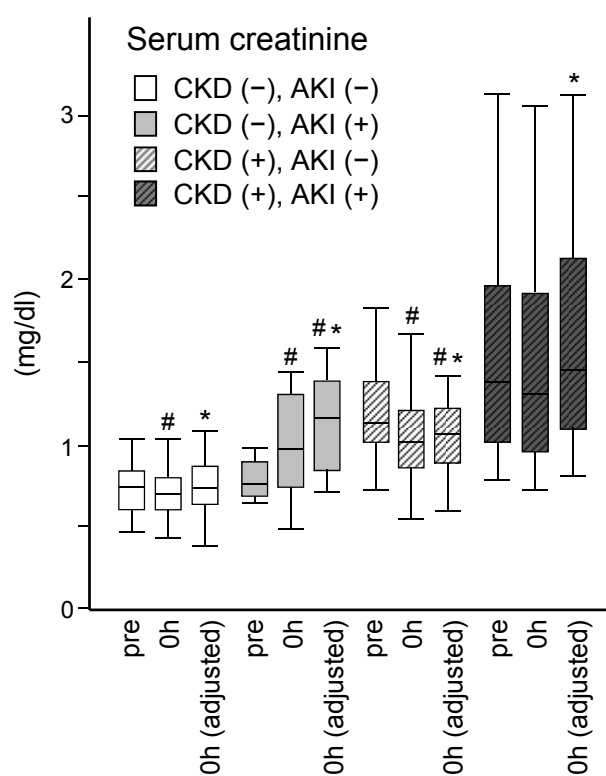**B**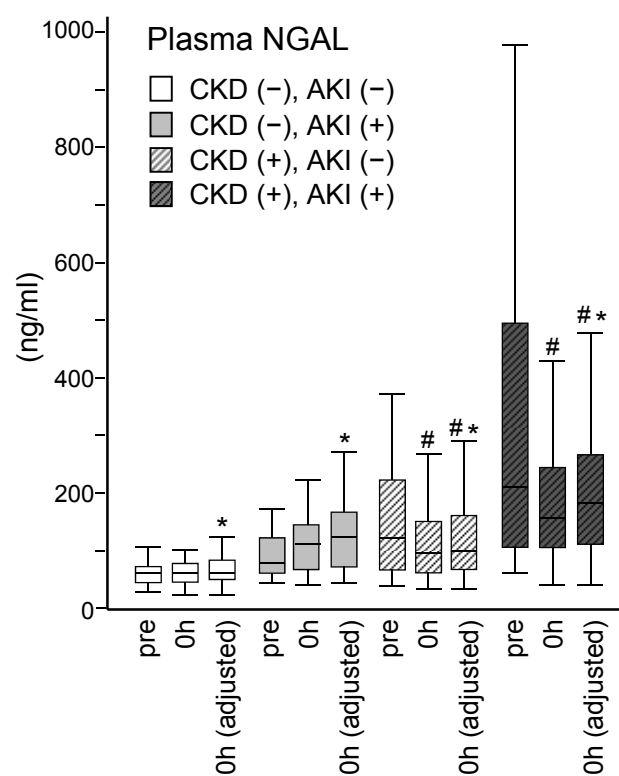

Supplementary figure 3

Supplement: Additional file 3: Figure S3 — The following additional data are available with the online version of this paper. Dilution effect on serum creatinine and plasma NGAL at 0 h. Values of serum creatinine (A) and plasma NGAL (B) before surgery and at 0 h (with and without adjustment by fluid accumulation) are shown (CKD(-)AKI(-) (n = 54), CKD(-)AKI(+) (n = 20), CKD(+)AKI(-) (n = 39), and CKD(+)AKI(+) (n = 33)). #, P <0.05 vs. before surgery (pre), *, P <0.05 vs. 0 h. [file cc13104-S3.pdf]
